# Supplementary material for: Identification of the Effects of Aspirin and Sulindac Sulfide on the Inhibition of HMGA2-Mediated Oncogenic Capacities in Colorectal Cancer
Source: Molecules. 2020 Aug 22;25(17):3826. doi: 10.3390/molecules25173826 (PMC7504004; doi:10.3390/molecules25173826)
Supplement: Supplementary file 1 [file molecules-25-03826-s001.pdf]

Supplemental Material includes three Supplemental Tables

Identification of the Effects of Aspirin and Sulindac Sulfide on the Inhibition of HMGA2-Mediated Oncogenic Capacities in Colorectal Cancer

Titus Ime Ekanem [1,2,†], Wei-Lun Tsai [3,†], Yi-Hsuan Lin [4], Wan-Qian Tan [3], Hsin-Yi Chang [5], Tsui-Chin Huang [3,6], Hsin-Yi Chen [3,6,*] and Kuen-Haur Lee [3,6,7,8,*]

1 Ph.D. Program for Cancer Molecular Biology and Drug Discovery, College of Medical Science and Technology, Taipei Medical University and Academia Sinica, Taipei 11031, Taiwan; titusekanem@yahoo.com

2 Department of Hematology, University of Uyo, Uyo 520271, Nigeria

3 Graduate Institute of Cancer Biology and Drug Discovery, College of Medical Science and Technology, Taipei Medical University, Taipei 11031, Taiwan; mebar1995@gmail.com (W.L.T.); amanda_tan@tmu.edu.tw (W.Q.T.); tsuichin@tmu.edu.tw (T.C.H.)

4 Nutrition and Health Sciences, College of Nutrition, Taipei Medical University, Taipei 11031, Taiwan; ba06106066@tmu.edu.tw

5 Graduate Institute of Metabolism and Obesity Sciences, College of Nutrition, Taipei Medical University, Taipei, 11031, Taiwan; hsinyi.chang@tmu.edu.tw

6 Ph.D. Program for Cancer Molecular Biology and Drug Discovery, College of Medical Science and Technology, Taipei Medical University, Taipei 11031, Taiwan

7 TMU Research Center of Cancer Translational Medicine, Taipei Medical University, Taipei 11031, Taiwan

8 Cancer Center, Wan Fang Hospital, Taipei Medical University 11696, Taipei, Taiwan

* Correspondence:hyichen@tmu.edu.tw (H.Y.C.); khlee@tmu.edu.tw (K.H.L.); Tel.: +886-2-26972035 (H.Y.C.); +886-2-26972035 (K.H.L.); Fax: +886-2-66387537 (H.Y.C.); +886-2-66387537 (K.H.L.)

† These authors contributed equally to this work

Supplementary Material

Supplementary Table S1 Top 10 chemical perturbagens with positive enrichment score for gene expression signature of knockdown of HMGA2

Rank	Drug	Description	Enrichment score
1	Prestwick-1082	Small molecule perturbation from the CMAP	0.891
2	Sulindac	Nonsteroidal anti-inflammatory drug (NSAID) of the arylalkanoic acid class	0.851
3	Cloxacillin	An antibiotic useful for the treatment of a number of bacterial infections	0.834
4	Iopromide	A molecule used as a contrast medium	0.813
5	Pivmecillinam	An extended-spectrum penicillin antibiotic	0.808
6	Benzocaine	An ester local anesthetic	0.799
7	Antazoline	1st generation antihistamine	0.737
8	Rilmenidine	A prescription medication for the treatment of hypertension	0.736
9	Penbutolol	A medication in the class of beta blockers	0.734
10	Prestwick-857	Small molecule perturbation	0.732

Supplementary Table S2 Top 10 chemical perturbagens with negative enrichment score for gene expression signature of overexpression of HMGA

**Rank**	**Drug**	**Description**	**Enrichment score**
1	Vemurafenib	MAP kinase kinasekinase (MAP3K) inhibitor	-0.803
2	Aspirin	Nonsteroidal anti-inflammatory drug (NSAID)	-0.735
3	PD-0325901	Mitogen-activated protein (MAP) kinase inhibitor	-0.701
4	Avrainvillamide-analog-3	Nucleophosmin inhibitor	-0.671
5	YC-1	Hypoxia inducible factor inhibitor	-0.586
6	4,5-dianilinophthalimide	Epidermal growth factor receptor (EGFR) inhibitor	-0.562
7	AS-605240	Phosphatidylinositol 3-kinase (PI3K) inhibitor	-0.562
8	Fulvestrant	Estrogen receptor antagonist	-0.544
9	Carbenoxolone	11-beta hydroxysteroid dehydrogenase inhibitor	-0.530
10	Bucladesine	Adenosine receptor agonist	-0.529

Supplementary Table S3 67 overlapping genes were identified from intersection between knockdown of HMGA2 or overexpression of HMGA2 from GSEA

Rank	Gene Name	Full Nane
1	ADORA2B	Adenosine A2b Receptor
2	AQP9	Aquaporin 9
3	ATP2A2	ATPase Sarcoplasmic/Endoplasmic Reticulum Ca^{2+} Transporting 2
4	AXL	AXL Receptor Tyrosine Kinase
5	BEST1	Bestrophin-1
6	C3AR1	Complement C3a Receptor 1
7	C5AR1	Complement C5a Receptor 1
8	CCL2	C-C Motif Chemokine Ligand 2
9	CCL22	C-C Motif Chemokine Ligand 22
10	CCR7	C-C Motif Chemokine Receptor 7
11	CCRL2	C-C Motif Chemokine Receptor Like 2
12	CD14	Monocyte Differentiation Antigen CD14
13	CD55	CD55 Antigen
14	CD69	Early Activation Antigen CD69
15	CD70	Surface Antigen CD70
16	CDKN1A	Cyclin Dependent Kinase Inhibitor 1A
17	CHST2	Carbohydrate Sulfotransferase 2
18	CMKLR1	Chemerin Chemokine-Like Receptor 1
19	CSF1	Macrophage Colony-Stimulating Factor 1
20	CXCR6	C-X-C Motif Chemokine Receptor 6
21	CYBB	Cytochrome B-245 Beta Chain
22	EMP3	Epithelial membrane protein 3
23	FPR1	Formyl Peptide Receptor 1
24	GNA15	G Protein Subunit Alpha 15
25	GPR132	G Protein-Coupled Receptor 132
26	HRH1	Histamine Receptor H1
27	IL10	Interleukin 10
28	IL10RA	Interleukin 10 Receptor Subunit Alpha
29	IL18RAP	Interleukin 18 Receptor Accessory Protein
30	IL1B	Interleukin 1 Beta
31	IL1R1	Interleukin 1 Receptor Type 1
32	IL4R	Interleukin 4 Receptor
33	IL6	Interleukin 6
34	IL7R	Interleukin 7 receptor

35	IRAK2	Interleukin 1 Receptor Associated Kinase 2
36	IRF7	Interferon Regulatory Factor 7
37	ITGB3	Integrin Subunit Beta 3
38	KCNA3	Potassium Voltage-Gated Channel Subfamily A Member 3
39	KCNMB2	Potassium Calcium-Activated Channel Subfamily M Regulatory Beta Subunit 2
40	KIF1B	Kinesin Family Member 1B
41	LCP2	Lymphocyte Cytosolic Protein 2
42	LDLR	Low Density Lipoprotein Receptor
43	LIF	LIF Interleukin 6 Family Cytokine
44	LTA	Lymphotoxin Alpha
45	LYN	LYN Proto-Oncogene, Src Family Tyrosine Kinase
46	MARCO	Macrophage Receptor With Collagenous Structure
47	MEFV	MEFV Innate Immuity Regulator, Pyrin
48	MXD1	MAX Dimerization Protein 1
49	NOD2	Nucleotide Binding Oligomerization Domain Containing 2
50	NPFFR2	Neuropeptide FF Receptor 2
51	OSMR	Oncostatin M Receptor
52	P2RX7	Purinergic Receptor P2X 7
53	PDE4B	Phosphodiesterase 4B
54	PIK3R5	Phosphoinositide-3-Kinase Regulatory Subunit 5
55	PTAFR	Platelet Activating Factor Receptor
56	PTGIR	Prostaglandin I2 Receptor
57	PTPRE	Protein Tyrosine Phosphatase Receptor Type E
58	RGS16	Regulator Of G Protein Signaling 16
59	RHOG	Ras Homolog Family Member G
60	RTP4	Receptor Transporter Protein 4
61	STAB1	Stabilin 1
62	TACR1	Tachykinin Receptor 1
63	TIMP1	Tissue Inhibitor Of Metalloproteinases 1
64	TLR1	Toll-Like Receptor 1
65	TNFRSF1B	TNF Receptor Superfamily Member 1B

66	TNFSF9	Tumor Necrosis Factor Ligand Superfamily Member 9
67	TPBG	Trophoblast Glycoprotein
